# Study on the graft modification mechanism of macroporous silica gel surface based on silane coupling agent vinyl triethoxysilane

**DOI:** 10.1039/d1ra04296c

**Published:** 2021-07-20

**Authors:** Zheng Wang, Mei-chen Liu, Zhi-yuan Chang, Hui-bo Li

**Affiliations:** Department of Radiochemistry, China Institute of Atomic Energy Beijing 102413 China hb0012@sina.com

## Abstract

In this research, the graft modification mechanism of coupling agent vinyl triethoxysilane (KH-151) to macroporous silica gel was studied using a combination of multiple methods. SEM, FTIR, RAMAN, NMR and XPS were used to characterize the silica gel before and after grafting, determining the mechanism of the grafting reaction of the silane coupling agent. On this basis, the grafting rate of the coupling agent was accurately calculated by TGA weight loss. From the results of characterization, it can be seen that the coupling agent molecules have two connection types on the surface of silica gel, and the ratio of the two types is 43.51% and 56.49% respectively. The influences of hydration degree of the silica gel, coupling agent dosage and reaction temperature on the grafting rate were explored, and the optimal reaction conditions for the modification of macroporous silica gel were determined by the coupling agent through orthogonal experiments, that is, the hydration degree of silica gel of 10%, a coupling agent dosage of 12 mL, and a reaction temperature of 80 °C. Under optimal reaction conditions, the average grafting rate of the coupling agent vinyl triethoxysilane (KH-151) on macroporous silica gel is as high as 91.03%.

## Abstract 2

Study on the mechanism of the alkylation reaction on the surface of porous silica gel.

## Graphical abstract

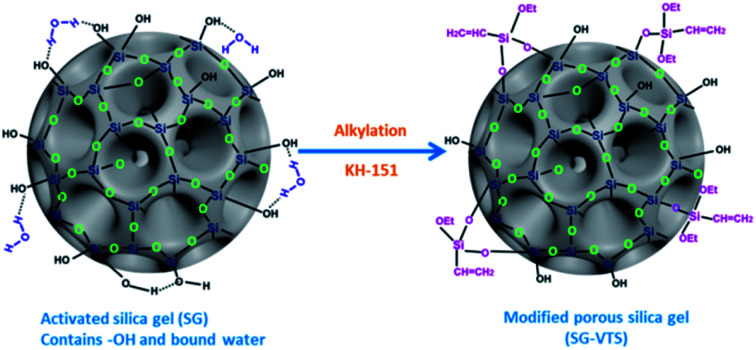

## Introduction

Porous silica gel is an non-crystal substance. With its good physical and chemical stability, especially high mechanical strength, high radiation stability, acid resistance, porous silica gel has been widely used in adsorbent carriers, catalyst carriers, biomedicine and other fields.^1,2^ However, as an inorganic matrix, it is difficult for silica gel to react directly with organic functional materials. Therefore, it is crucial to functionally modify it. The solution of many problems is inseparable from the functional modification of silica gel matrices, and the functional modification of silica gel surfaces has become one of the important topics in the field of silica gel materials.

In previous studies, people have developed many methods of silica gel modification, and among them, chemical modification methods dominate.^3^ Chemical modification refers to the use of a modifier to chemically react with the –OH on the surface of the silica gel, so that the modifier is chemically bonded to the surface of the silica gel. In this process, the inherent characteristics of silica gel do not change, such as mechanical strength, thermal stability, acid resistance, *etc.*^4,5^ Commonly used methods for chemical modification of silica gel surfaces mainly include the alcohol esterification method and coupling agent method.^6^ The alcohol esterification method refers to the dehydration and condensation reaction between the –OH on the surface of the silica gel and a fatty alcohol under the conditions of specific pressure and temperature to generate water and silicone grease, thereby grafting the required groups to the surface of the silica gel.^7–10^ Ossenkamp *et al.* used *n*-octanol as esterification agent to modify a nano silica gel, and the obtained material had excellent lipophilic and hydrophobic properties.^11^ However, there are fewer types of modifiers used in the alcohol esterification method, which makes the application range of this method not wide.

**Fig. 1:**
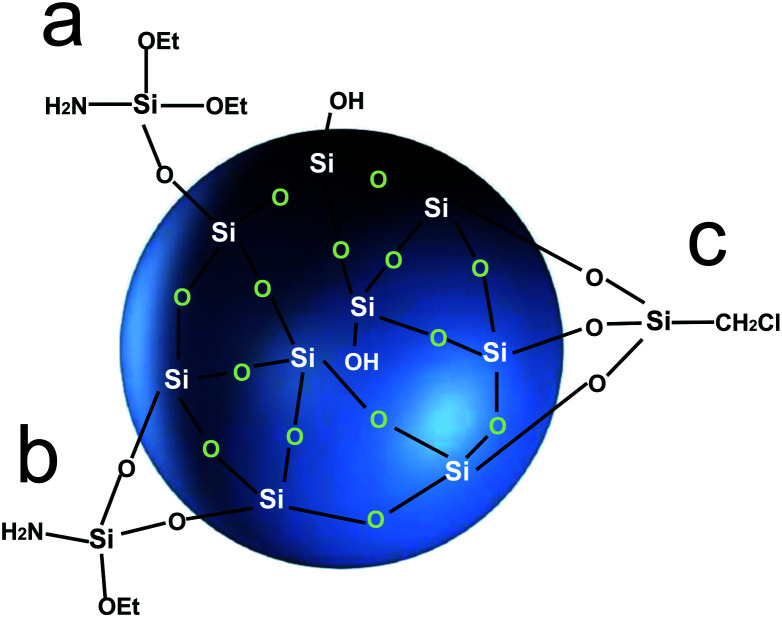
Coupling agent connection form.

The coupling agent method is the most widely used method for surface modification of silica gel. The silane coupling agent can be represented by the chemical formula R-SiX_3_. Among them, X is a functional group that can be hydrolyzed, such as –OCH_3_ or –OCH_2_CH_3_, which can react with the –OH on silica gel in the way of dehydration and condensation; the other group R is an organic reactive group that does not hydrolyze, such as C=C, –NH_2_ and –CH_2_Cl, *etc.*, which can easily react with other organic groups, and can graft functional materials onto the surface of the silica gel.^12^ Research studies on the reaction mechanism and grafting effect of coupling agents have been reported in the literature. Wachsmann *et al.* used amino-type coupling agents to modify silica gel; they believed that only one –OCH_2_CH_3_ on the coupling agent participated in the reaction, as shown in Fig. 1a;^13,14^ Pirkle *et al.* used the synthesis of chiral stationary phases to separate amino acid enantiomers; there are two –OCH_2_CH_3_ groups on the coupling agent and the silica gel has a bonding reaction, as shown in Fig. 1b;^15,16^ Mayani *et al.* in their research believed that the three –OCH_2_CH_3_ groups on the coupling agent are all involved in the bonding reaction, resulting in the structure of the product shown in Fig. 1c.^17^ However, most of the previous research on this aspect was carried out as a small part of the synthesis process. Due to the single characterization method and rough analysis method, research results are not accurate, which indirectly leads to inaccurate calculation results of the grafting rate of the coupling agent. Surface modification, as a link between the past and next, is vital in the process of functionalization of silica gel materials. Therefore, it is necessary to study the reaction mechanism of coupled-link modified silica gel with a more accurate method.

In this study, we use macroporous silica gel (SG) with a pore size of about 10 nm as the matrix; after sieving and acidification pretreatment, the surface is chemically modified with coupling agent vinyl triethoxysilane (KH-151), yielding synthetic coupling agent modified silica gel (SG-VTS). The study is divided into two parts. First, we use SEM, FTIR, Raman, NMR and XPS to characterize silica gel before and after grafting, and determine the mechanism of the grafting reaction. On this basis, the grafting rate of the coupling agent was determined by the TGA weight loss change. Second, we study the changing law of grafting rate of silica gel with different particle sizes. The influence of the single factor of coupling agent dosage, temperature and silica hydration degree on the grafting rate of coupling agent was explored, and the optimal reaction conditions of coupling agent modified silica gel were determined by orthogonal experiment.^18,19^ This work has explored a new path for the in-depth, detailed and comprehensive research on surface chemical modification of silica gel, and provided a theoretical basis for chemical modification of the surface of silica gel based on coupling agents.

## Material and methods

### Materials

Macroporous silica gel (pore size 8 nm, particle size 100–200 mesh) is produced by Qingdao Kaibang High-tech Materials limited company. Absolute ethyl alcohol, HNO_3_ (excellent grade), xylene (analytical grade), vinyl triethoxysilane (KH-151) (98%) were purchased from Aladdin Reagent (Shanghai) limited company. Deionized water was prepared by ET461275 ultra pure water machine (Lovibond, Germany).

### Preparation of KH-151 modified silica gel (SG-VTS)

After washing with deionized water, immerse the macroporous silica gel in 1 mol L^−1^ HNO_3_ and activate for 6 hours to increase the –OH on the surface of the silica gel.^20,21^ The activated process is carried out in an ultrasonic cleaner (KQ-300E, Shumei, China), and the temperature is set to 60 °C. Then, the silica gel was washed with deionized water and placed in an oven for 24 hours to obtain activated silica gel (SG). The coupling agent KH-151 (98%) was placed in R-300 rotary evaporator (Buchi, Switzerland), and the polymerization inhibitor was removed under vacuum at 80 °C. The activated silica gel (SG) and xylene were mixed and placed in DDL-2000 reactor (EYELA, Japan) and stirred for 15 minutes, then KH-151 was added, and the mixture was stirred and refluxed for 24 hours at a constant temperature. After the grafting reaction, the product was washed three times with ethanol and dried under vacuum at 60 °C for 24 hours to obtain modified silica gel (SG-VTS).

### Characterization of SG and SG-VTS

The microstructures of the SG and SG-VTS were imaged on S-4800 scanning electron microscopy (Hitachi, Japan). Fourier transform infrared (FTIR) spectra were obtained on a IS50 FTIR spectrometer (Nicolet, USA). Raman spectra were obtained on inVia Raman microscope (Renishaw, UK). Nuclear Magnetic Resonance (NMR) spectra of the SG and SG-VTS were recorded on Biospin Avance III spectrometer (Bruker, ^29^Si, 600 MHz, USA). Escalab 250Xi X-ray photoelectron spectrometer (XPS) (Thermo Scientific, USA) was used to measure surface element content and surface functional groups. SG and SG-VTS were thermogravimetrically analyzed in DSC3+ thermogravimetric analyzer (Mettler Toledo, Switzerland). The specific surface area and pore structure of SG and SG-VTS were measured on SSA-3000 specific surface area and porosity analyser (Builder, China). The specific surface area was calculated by the Brunauer–Emmett–Teller (BET) method. Pore diameter were calculated by Barrett–Joyner–Halenda (BJH) method.^22,23^

### Calculation method for grafting rate

The grafting rate of coupling agent KH-151 on SG is calculated by formula (1):1
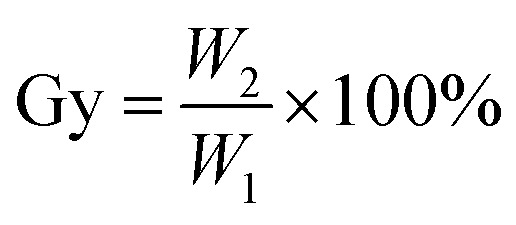
where Gy (%) is the grafting rate, namely the proportion of –OH involved in the grafting reaction, *W*_1_ (mmol g^−1^) is the total amount of –OH before the grafting reaction, and *W*_2_ (mmol g^−1^) is the hydroxyl groups participating in the grafting reaction quantity. *W*_1_ can be calculated by the thermogravimetric analysis of SG. The –OH on the surface of silica gel will undergo dehydration and condensation during heating. The general reaction is as follows (2):2
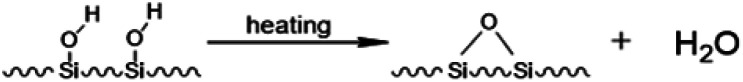


According to the reaction, the calculation formula for the surface –OH content of SG is as follows (3):3
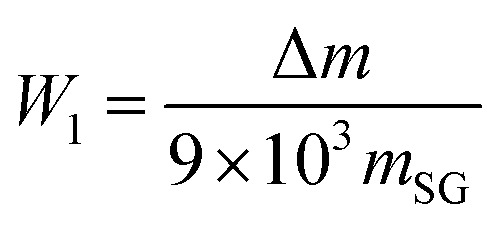
where Δ*m* is weight loss of SG, and *m*_SG_ is original weight of silica gel.

Since the coupling agent KH-151 may have different connection methods on the surface of silica gel, *W*_2_ can be calculated by analyzing the reaction mechanism in the characterization combined with the weight loss of the organic components in the modified silica gel. Refer to the formula (4) in section Characterization of SG and SG-VTS for details.

### Batch experiments

(i) Sieving the macroporous silica gel step by step: 100–120 mesh, 120–140 mesh, 140–160 mesh, 160–180 mesh, 180–200 mesh. Other conditions: SG hydration degree 7%, reaction temperature 60 °C and reaction time 24 h.

(ii) Set the dosage of coupling agent KH-151: 1 mL, 2 mL, 3 mL, 4 mL, 5 mL, 6 mL, 8 mL, 10 mL, 12 mL, 15 mL. Other conditions: SG particle size 180–200 mesh, SG hydration degree 7%, reaction temperature 60 °C and reaction time 24 h.

(iii) Set the reaction temperature gradient: 30 °C, 40 °C, 50 °C, 60 °C, 70 °C, 80 °C, 90 °C. Other conditions: SG particle size 180–200 mesh, SG hydration degree 7% and reaction time 24 h.

(iv) Six parts of SG were placed in beaker respectively, using the moisture in the air to hydrate naturally and weighing them every 1 hour. When the weight gain reaches 3%, 5%, 7%, 10%, 12%, 15%, respectively, SG was quickly added to the reactor, refluxed and stirred for grafting reaction. Other conditions: xylene solvent 30 mL, coupling agent KH-151 10 mL, SG particle size 180–200 mesh, reaction temperature 60 °C and reaction time 24 h.

After each set of reactions, the product was washed three times with ethanol and dried under vacuum at 60 °C for 24 hours to obtain modified silica gel SG-VTS. SG and SG-VTS are respectively subjected to thermogravimetric analysis to determine weight loss. Use formulas (1), (3) and (4) to calculate grafting rate.

### Orthogonal test

Based on single factor test, the silica gel hydration degree (*A*), reaction temperature (*B*) and silane coupling agent KH-151 dosage (*C*) were selected as the main influencing factors, and the L_9_(3^4^) orthogonal test was performed with the coupling agent KH-151 grafting rate as the index to determine the optimum preparation process for the grafting reaction. The levels of orthogonal factors are shown in Table 1. SPSS software is used to perform orthogonal test variance analysis.

**Table 1:** Orthogonal test level distribution

Factor	Level		
	1	2	3
Hydration degree (*A*)/%	8	10	12
Temperature (*B*)/°C	60	70	80
Coupling agent dosage (*C*)/mL	8	10	12

**Fig. 2:**
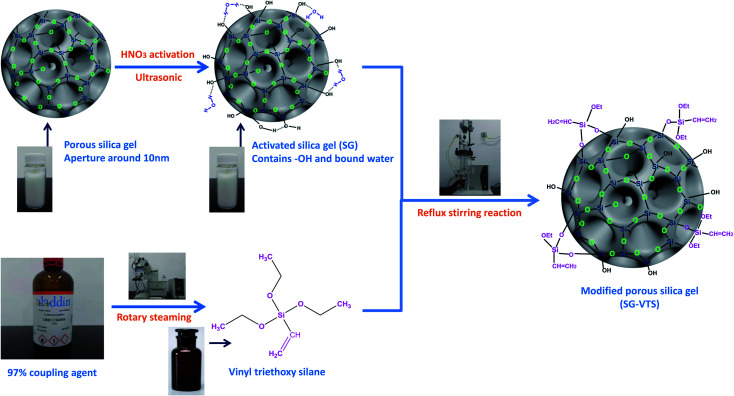
Preparation process of SG-VTS.

The preparation processes of SG and SG-VTS are shown in Fig. 2.

## Results and discussion

### Characterization of SG and SG-VTS

**Fig. 3:**
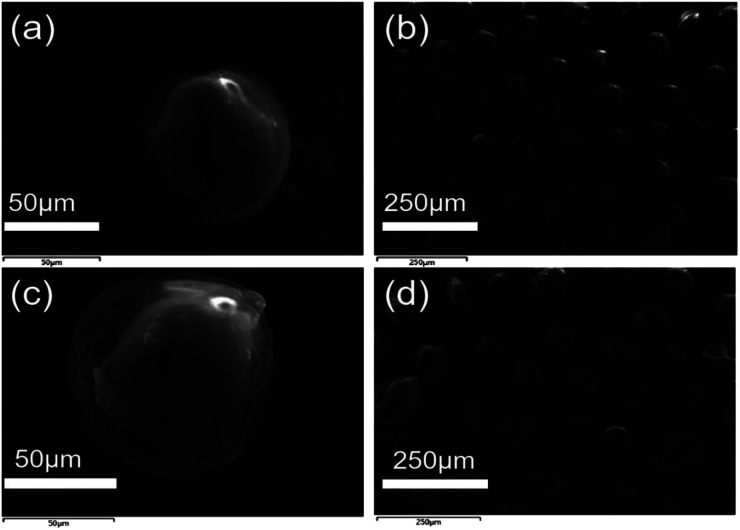
SEM images of SG (a and b) and SG-VTS (c and d).

**Fig. 4:**
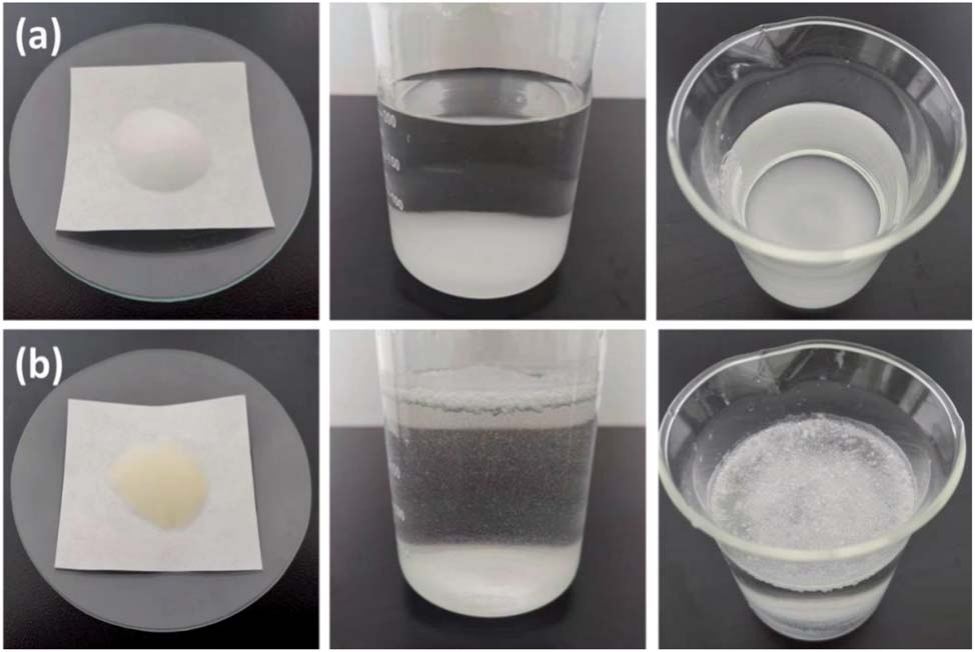
Comparison of hydrophilicity between SG (a) and SG-VTS (b).

The SEM images of SG (a and b) and SG-VTS (c and d) are shown in Fig. 3. It can be seen from Fig. 3a and b that the surface of SG is relatively smooth, while the surface of SG-VTS is obviously uneven, as shown in Fig. 3c and d, which shows that KH-151 has been successfully modified to the surface of silica gel. In addition, there are a few cracks on the surface of SG-VTS. This may be because silica gel is a strong water-absorbing substance and the surface is wet. However, there are a large number of organic groups on the surface after grafting, which increases the hydrophobicity and reduces the amount of moisture to cause the surface to dry.^24^ The difference in hydrophilicity between SG and SG-VTS can be also obviously seen from Fig. 4. For SG (Fig. 4a), silica gel particles all sink to the bottom in deionized water, however, SG-VTS particles (Fig. 4b) are mostly suspended or floated in water, which is due to the existence of a large number of hydrophobic groups (–CH=CH_2_, –CH_2_CH_3_) on the surface.

**Fig. 5:**
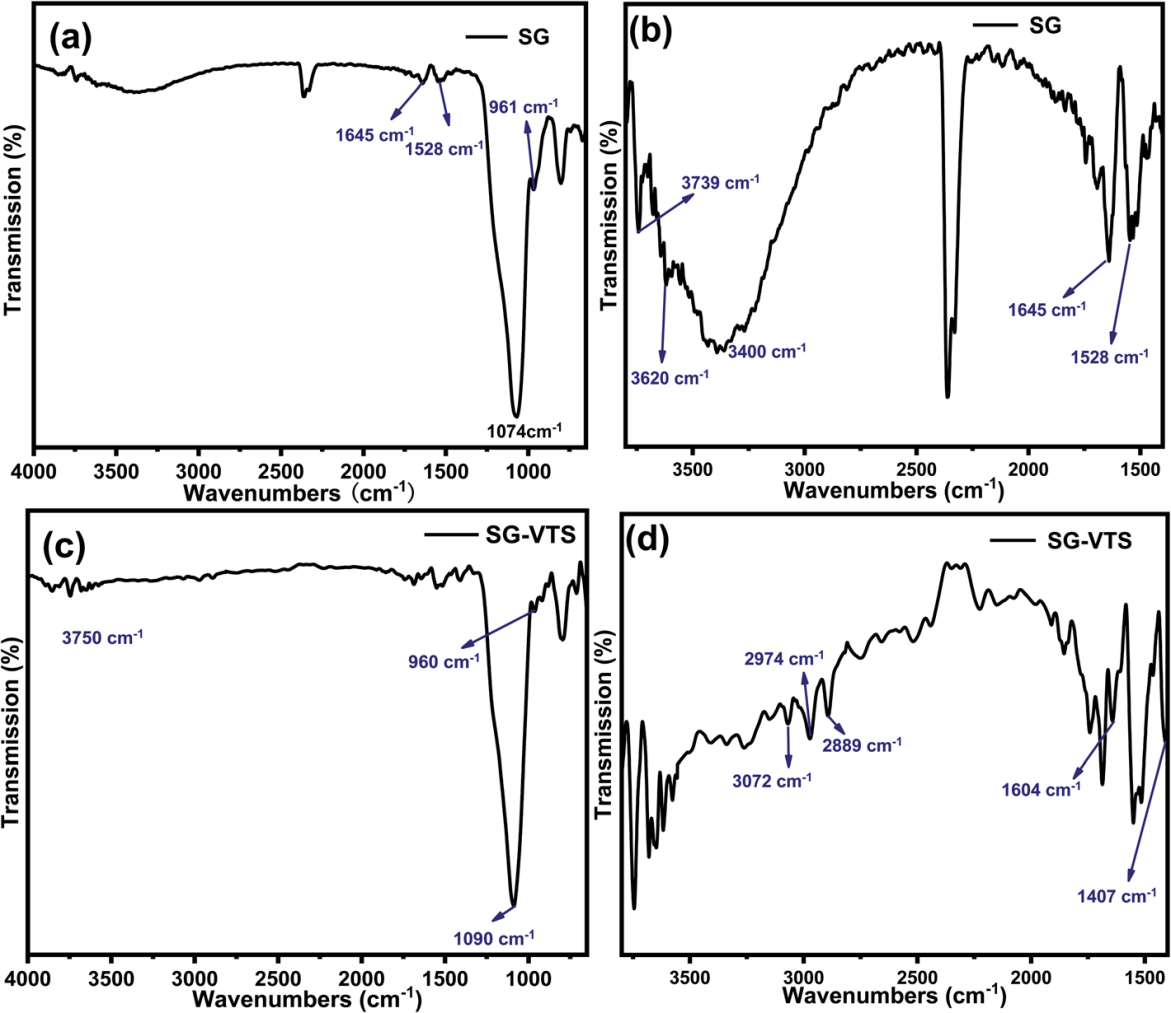
FTIR spectra of silica gel before (SG) (a and b) and after (SG-VTS) (c and d) grafting.

Fig. 5 exhibit FTIR of the SG and SG-VTS. For SG, in Fig. 5a, 961 cm^−1^ is the flexural vibration peak of –OH, 1528 cm^−1^ is the in-plane deformation vibration peak of –OH and 1645 cm^−1^ is H_2_O flexural vibration peak.^25,26^ In Fig. 5b (3000–4000 cm^−1^ enlarged part of Fig. 4a), the broad peak around 3400 cm^−1^ is the stretching vibration peak of the double-base –OH and H_2_O, 3620 cm^−1^ is the stretching vibration peak with hydrogen-bonded –OH and 3739 cm^−1^ is the stretching vibration peak of the free-type –OH.^27^Fig. 5a and b showed that there are three different types of –OH on the surface of SG, namely free-type –OH, hydrogen-bonded –OH and double-base –OH. In addition, there is a certain amount of bound water on the surface of SG.

For SG-VTS, in Fig. 5c, compared with SG, due to the reduction of surface bound water, the bending vibration peak of water at 1645 cm^−1^ and the stretching vibration peak of water around 3400 cm^−1^ disappear, but the peak at 3400 cm^−1^ is also double-base –OH peak, which indicates that the double-base –OH is involved in the grafting reaction. Due to the decrease in the total number of –OH, the flexural vibration peaks of –OH at 961 cm^−1^ and the stretching vibration peaks of free-type –OH at 3750 cm^−1^ were significantly weakened. In Fig. 5d (1400–3000 cm^−1^ enlarged part of Fig. 5a), 3072 cm^−1^ is the stretching vibration peak of C–H on C=C, 2974 cm^−1^ and 2889 cm^−1^ are the stretching vibration peaks of C–H in –CH_3_, 1604 cm^−1^ is the stretching vibration peak of C=C, however, the corresponding position in SG does not have this characteristic peak. 1407 cm^−1^ is the in-plane bending vibration peak of C–H in –CH_3_.^28–30^ The above analysis shows that the characteristic peaks of the groups in the coupling agent KH-151 are all present in the SG-VTS spectrum, which indicates that the coupling agent KH-151 has been successfully grafted on the surface of the silica gel.

**Fig. 6:**
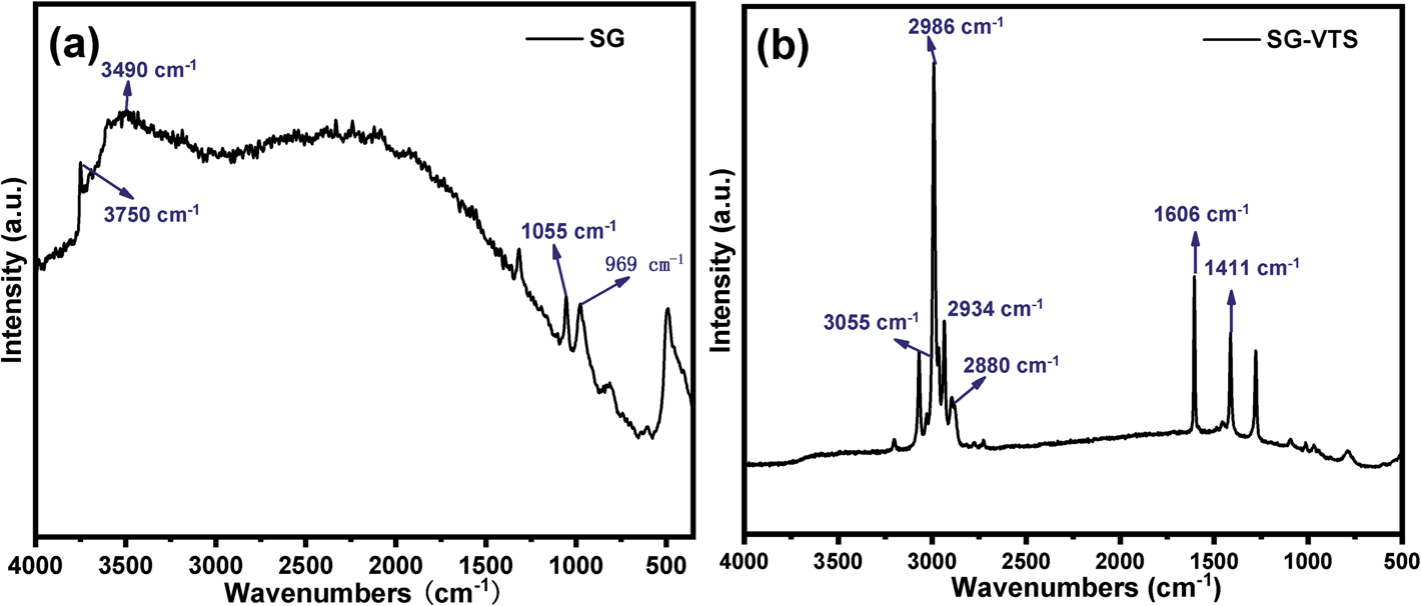
Raman spectra of SG (a) and SG-VTS (b).

The Raman spectroscopies of SG and SG-VTS are shown in Fig. 6. As shown in Fig. 6a, for SG, 3750 cm^−1^ is the stretching vibration peak of free-type –OH, 3490 cm^−1^ is the stretching vibration peak of the double-base –OH and 969 cm^−1^ is the flexural vibration peak of –OH.^31^ For SG-VTS, in Fig. 6b, the characteristic peak of –OH in the silica gel disappears. 1606 cm^−1^ is the stretching vibration peak of C=C, 3055 cm^−1^ is the stretching vibration peak of C–H on C=C, 1411 cm^−1^ is the in-plane bending vibration peak of C–H in –CH_3_, 2986 cm^−1^ and 2880 cm^−1^ is the stretching vibration peak of C–H in –CH_3_ and 2934 cm^−1^ is the stretching vibration peak of C–H in –CH_2_–.^32,33^ The results of Raman spectroscopy further show that the coupling agent KH-151 has been successfully grafted on the surface of silica gel.

**Fig. 7:**
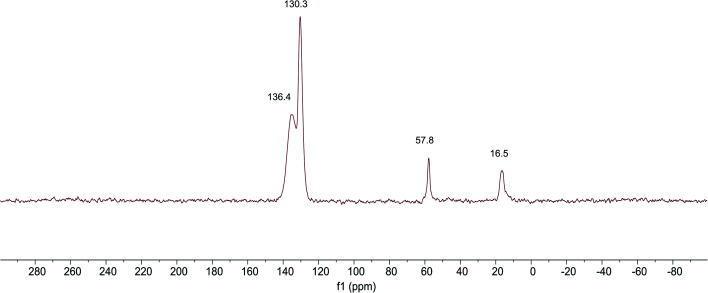
^13^C NMR spectra of SG-VTS.

The ^13^C NMR spectra of SG-VTS is shown in Fig. 7, in which 16.4 ppm is the absorption peak of C corresponding to the –CH_3_, 57.8 ppm is the absorption peak of C corresponding to the –OCH_2_–. In addition, 130.4 ppm is the absorption peak of C corresponding to the =CH_2_ and 135.4 ppm is the absorption peak of C corresponding to the –CH= . The analysis results of ^13^C NMR are consistent with those of IR and Raman, which shows the existence of –OCH_2_CH_3_ and –CH=CH_2_ functional groups in SG-VTS.

**Fig. 8:**
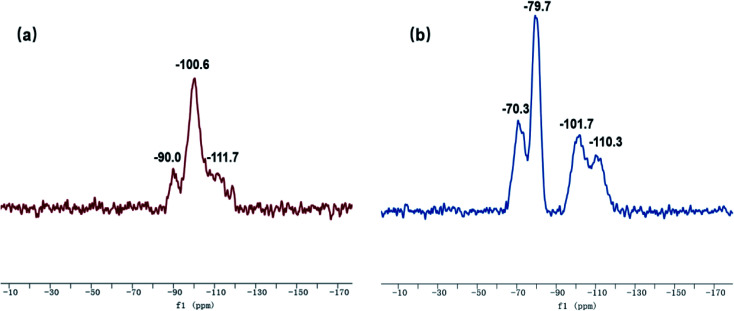
^29^Si-MAS NMR spectra of SG (a) and SG-VTS (b).

**Fig. 9:**
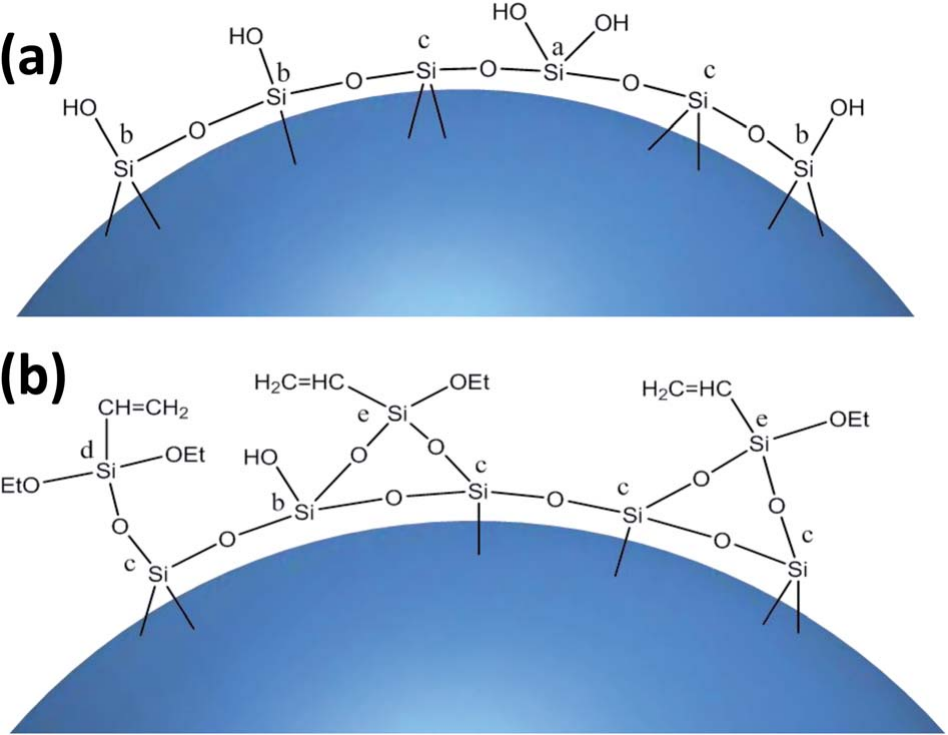
Surface morphology of SG (a) and SG-VTS (b).

The ^29^Si-MAS NMR spectra of SG and SG-VTS are shown in Fig. 8. Fig. 8a is the ^29^Si-MAS NMR spectrum of unmodified silica gel SG, in which −90.0 ppm is the absorption peak of Si corresponding to the double-base –OH, −100.6 ppm is the absorption peak of Si corresponding to the independent –OH (including free-type –OH and hydrogen-bonded –OH), and −111.7 ppm is absorption peak of Si corresponding to siloxane groups Si-(OSi)_4_, in addition, there is no absorption peak of other types of Si.^34,35^ According to the ^29^Si-MAS NMR spectrum analysis of SG, the possible surface morphology of the original silica gel SG is shown in Fig. 9a.

Fig. 8b is a ^29^Si-MAS NMR spectrum of modified silica gel SG-VTS. Compared with the SG spectrogram, the absorption peak at −90.0 ppm disappeared, indicating that double-base –OH almost completely participated in the reaction. The intensity of the absorption peak at −101.7 ppm decreased, indicating that the independent –OH participated in the grafting reaction. However, the reduction in the intensity of the −101.7 ppm peak is relatively small, which may be due to the reaction of only one –OH in the double-base –OH, and the other one cannot undergo grafting reaction with the coupling agent due to steric hindrance and becomes a free-type –OH, which increases the content of independent –OH and increases the amount of corresponding Si. In addition, a large number of the original independent –OH participated in the reaction, increasing the number of Si corresponding to siloxane groups Si-(OSi)_4_, which enhanced the intensity of the absorption peak at −110.3 ppm. −70.3 ppm and −79.7 ppm are the absorption peaks of Si of coupling agent KH-151 with two different connection modes on the surface of the silica gel respectively. Among them, −70.3 ppm corresponds to coupling agent molecule with only one chemical bond connected to the silica body, and −79.7 ppm corresponds to coupling agent molecule with two chemical bonds connected to the silica body.^36,37^ According to the analysis, the possible surface morphology of silica gel SG-VTS after grafting is shown in Fig. 9b.

**Fig. 10:**
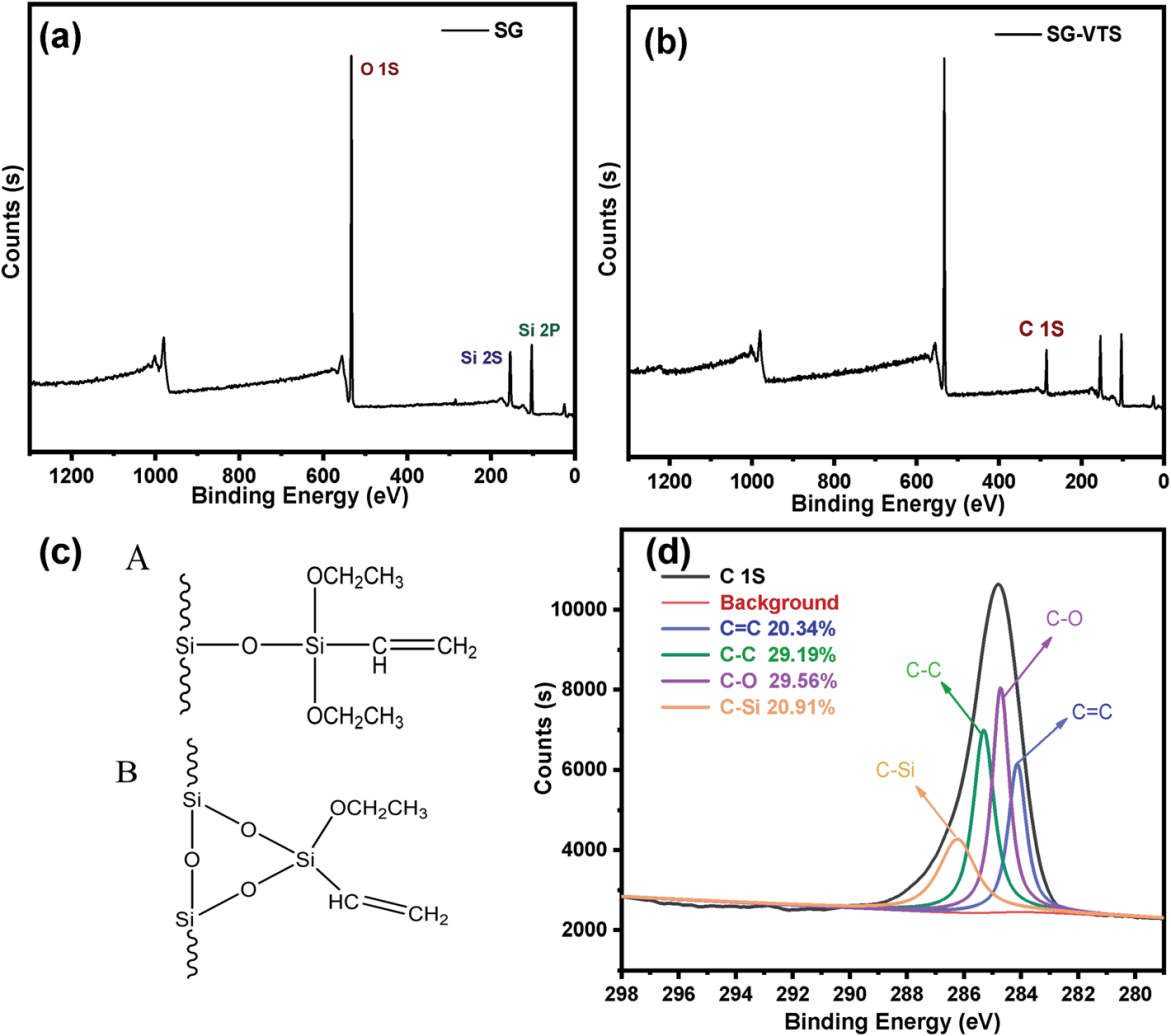
The XPS spectra of SG (a) and SG-VTS (b); Two connection types of coupling agent KH-151 (c); the deconvolution of C 1s spectra (d).

The XPS spectrogram of SG and SG-VTS are shown in Fig. 10. In Fig. 10a, SG only contains two elements: Si and O. Among them, the binding energy 103 eV is the Si 2p absorption peak, 153 eV is the Si 2s absorption peak, 533 eV is the O 1s absorption peak and 985 eV is the 0 KLL absorption peak of O.^38^ Compared with SG, in SG-VTS (Fig. 10b) spectrogram, in addition to the Si and O absorption peaks, there is also the C 1s absorption peak at 285 eV.^39^

According to the NMR analysis results, there are two connection types to connect coupling agent KH-151 to the silica gel, as shown in Fig. 10c. In order to explore the proportions of different connection types of coupling agents, we fitted the C 1s absorption peak. In SG-VTS, the number ratio of C=C, C–C, C–O, and C–Si is 1 : *n* : *n* : 1. If all KH-151 are connected in A type, the value of *n* is about 2; if all KH-151 are connected in B type, the value of *n* is about 1; if the two connection types coexist, then 1 < *n* < 2. Four deconvoluted peaks centered at 284.2 eV, 285.5 eV, 284.8 eV and 286.1 eV were assigned to C=C, C–C, C–O and C–Si groups respectively.^40^ The C 1s spectra were illustrated in Fig. 10d. According to the fitting results, the peak area ratios of the four groups are 20.342%, 29.187%, 29.559%, and 20.914% respectively, namely the content ratio of the four groups is 1 : 1.4351 : 1.4533 : 1.0280. According to the theoretical calculation based on the quantity ratio of C=C and C–C, the content of KH-151 connected by A type is 43.51%, and the content of KH-151 connected by B type is 56.49%. The proportions of the two connection methods combined with the subsequent thermogravimetric analysis will be used to accurately calculate the grafting rate of KH-151.

**Fig. 11:**
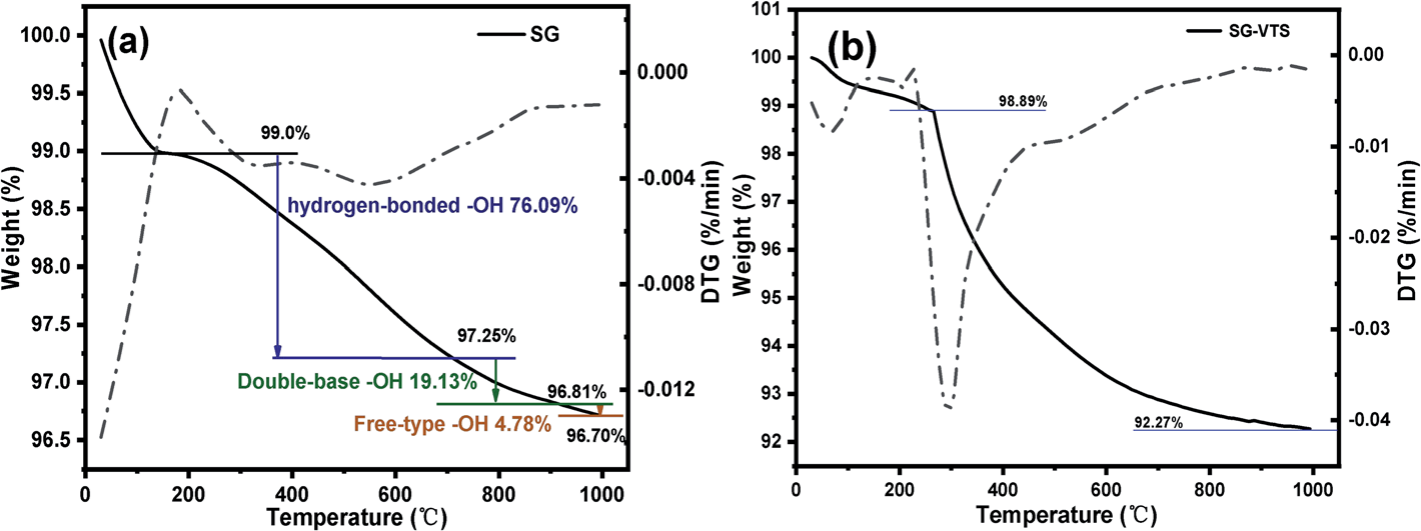
Thermogravimetric curve of SG (a) and SG-VTS (b).

The TG-DTG results of SG and SG-VTS are shown in Fig. 11. For SG (Fig. 11a), the weight loss in the range of 30–177 °C is due to the physical bound water evaporation of the surface of the silica gel.^41^ The weight loss in the range of 177–1000 °C is the dehydration condensation of the –OH on the silica gel surface, which accounts for about 2.3% of the total weight. Among them, 177–700 °C is the weight loss of hydrogen-bonded –OH, accounting for about 76.09% of the total –OH; 700–920 °C is the weight loss of double-base –OH, accounting for 19.13% of the total –OH; 920–1000 °C is the weight loss of free-type –OH, accounting for 4.78% of the total –OH.^42^ According to theoretical calculations, the –OH content on the surface of silica gel is about 2.54 mmol L^−1^. For SG-VTS (Fig. 11b), the weight loss in the range of 30–266 °C is the dissociation process of organic solvents such as xylene and ethanol on the surface of silica gel. 266–1000 °C is the decomposition process of –CH=CH_2_, –OCH_2_CH_3_ on the coupling agent molecule of the silica gel surface. According to the weight loss and the analysis results in NMR and XPS, the calculation formula (4) of the number of –OH participating in the grafting reaction can be obtained as follows:4
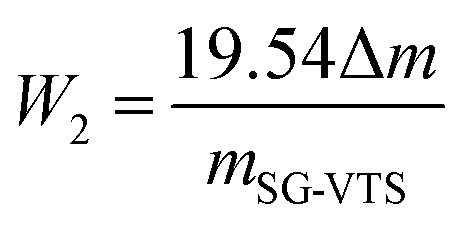
where *W*_2_ (mmol L^−1^) is the amount of –OH participating in the reaction, Δ*m* is the weight loss of the SG-VTS during the heating process and *m*_SG-VTS_ is the original weight of the SG-VTS.

**Fig. 12:**
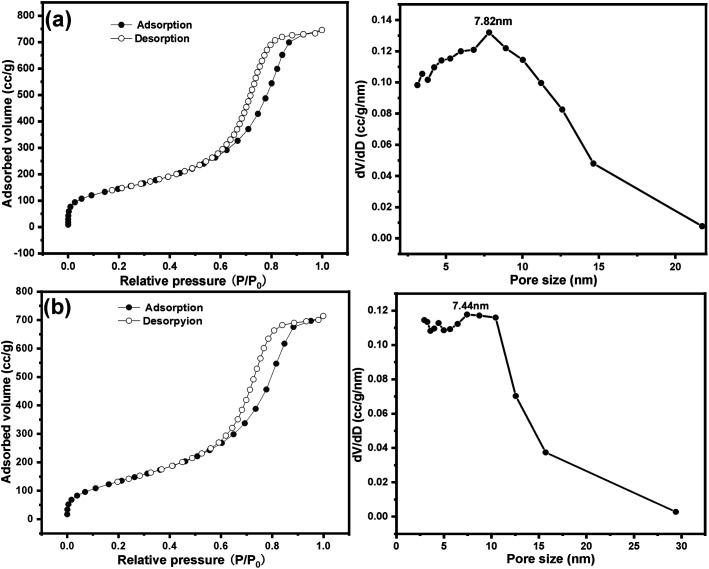
Nitrogen sorption isotherm and pore size distribution of SG (a) and SG-VTS (b).

Nitrogen sorption isotherm and pore size distribution of SG and SG-VTS are shown in Fig. 12. For SG, in Fig. 12a, the specific surface area and maximum probability aperture calculated by BET method and BJH method are 512.56 m^2^ g^−1^ and 7.82 nm respectively. After grafting reaction (Fig. 12b), the specific surface area and maximum probability aperture of SG-VTS are 431.82 m^2^ g^−1^ and 7.44 nm respectively. The above result indicates after being grafted by coupling agent KH-151, the porous silica gel material still has a relatively large pore size and specific surface area, which can be used for further functional modification.

### Research on law of grafting reaction and process optimization

#### Influence of silica gel particle size on the grafting result of KH-151

**Fig. 13:**
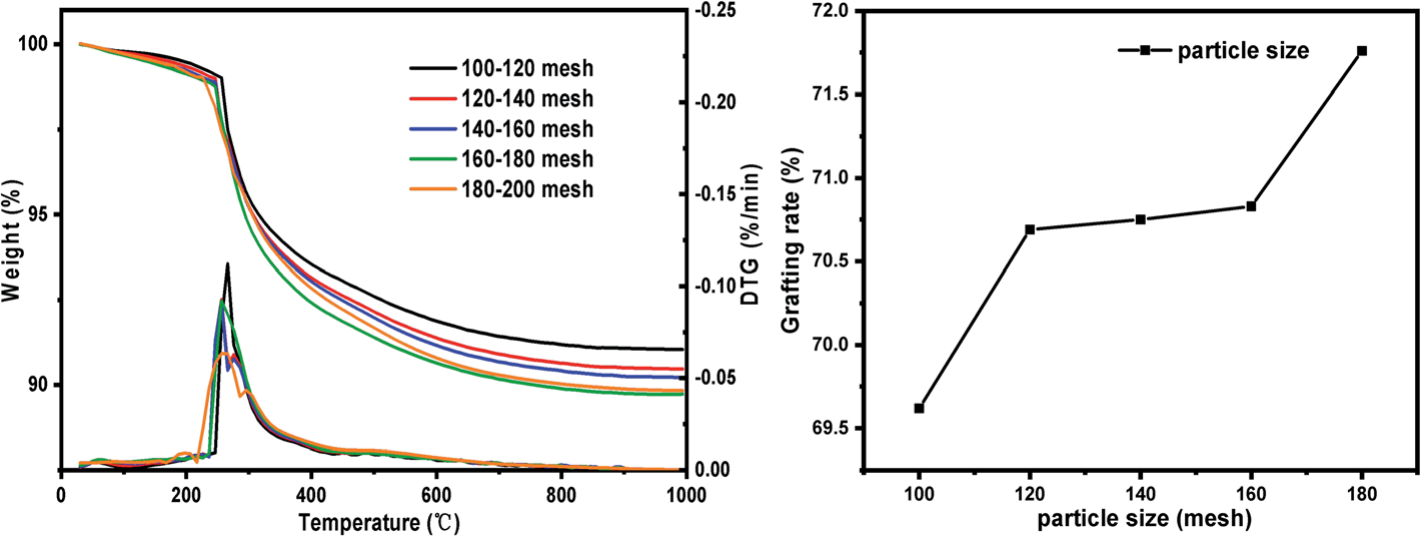
The influence of particle size change on grafting rate.

The laws of change in pore size, specific surface area and grafting rate of silica gel with different particle sizes before and after grafting were studied. The changes in pore size and specific surface area of silica gel with different particle sizes before and after grafting are shown in Table 2. According to the data in the table, the maximum probability aperture of the five particle sizes SG after sieving is about 8 nm, which shows that the preparation process of this kind of silica gel is relatively stable. As the particle size of SG decreases, the specific surface area and the number of surface –OH gradually increase. After grafting, the pore size and specific surface area of SG-VTS with different particle sizes are reduced. The effect of particle size on the grafting rate of coupling agent KH-151 is shown in Fig. 13. As the particle size decreases, the grafting rate of KH-151 gradually increases, which may be because the smaller particle size is more conducive to the coupling agent molecules to enter and fill the inside of the silica gel particle pores, thereby making contact with the silica gel surface more fully. Since 180–200 mesh silica gel particles have a large grafting rate, subsequent single factor experiments will be carried out using silica gel with this particle size range.

**Table 2:** Changes before and after grafting of different particle size silica gel

Particle size (mesh)	Pore size (nm) (SG)	Specific surface area (m^2^ g^−1^) (SG)	–OH quantity (mmol g^−1^)	Pore size (nm) (SG-VTS)	Specific surface area (m^2^ g^−1^) (SG-VTS)
100–120	7.95	435.81	2.41	7.6	403.59
120–140	7.97	466.36	2.53	7.57	410.47
140–160	8.16	478.81	2.65	7.55	423.47
160–180	7.98	485.41	2.67	7.55	423.79
180–200	7.82	512.56	2.69	7.44	431.82

#### Influence of the coupling agent KH-151 dosage on the grafting rate

**Fig. 14:**
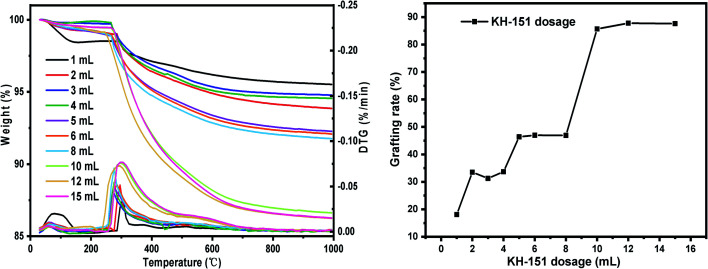
The influence of agent KH-151 dosage on the grafting rate.

The effects of different coupling agent dosage on the grafting rate are shown in Fig. 14. It can be seen from the change in the figure that as coupling agent dosage increases, the grafting rate shows an upward trend integrally. When KH-151 is less than 8 mL, as the dosage increases, the grafting rate rises slowly. And in the range of less than 8 mL, the grafting rate is all less than 50%. As the dosage of KH-151 continues to increase, the grafting rate rises sharply. When KH-151 is greater than 10 mL, the grafting rate basically tends to be stable, and the maximum grafting rate reaches 87.38%.

#### Influence of reaction temperature on grafting rate

**Fig. 15:**
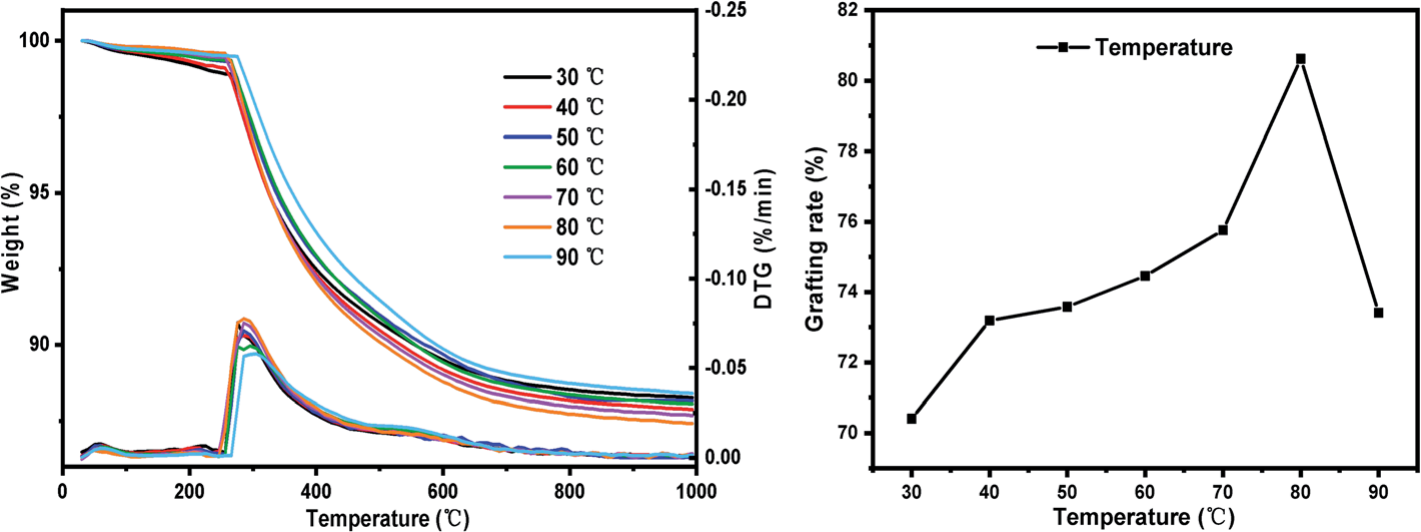
The influence of reaction temperature on the grafting rate.

The change law of grafting rate with reaction temperature is shown in Fig. 15. As the temperature increases, the grafting rate first increases and then decreases. In the range of 50–70 °C, as the temperature rises, the grafting rate increases slowly, which is because molecular thermal movement intensifies, the collision probability of KH-151 molecules with the silica gel surface increases, and the grafting rate follows increase. When the temperature reaches 80 °C, the grafting rate increases significantly, which is because the boiling point of ethanol is 78 °C. When the reaction temperature is greater than 78 °C, the ethanol produced can quickly volatilize from the reaction system, thereby promoting the progress of the reaction and grafting. Since the condensation reaction between the coupling agent and the –OH on the surface of the silica gel after hydrolysis is an exothermic reaction, when the temperature exceeds 80 °C, as the temperature rises, the equilibrium shifts to the side reaction direction and the grafting rate decreases.^43^

#### Influence of SG hydration degree on grafting rate

**Fig. 16:**
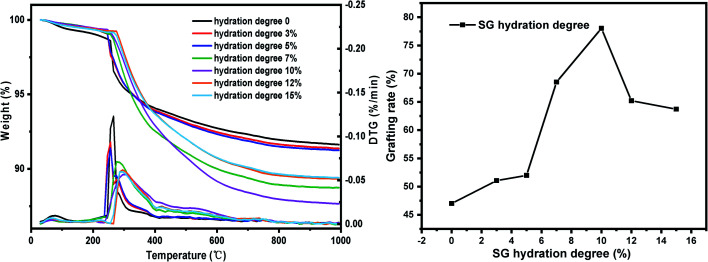
The influence of SG hydration degree on the grafting rate.

**Fig. 17:**
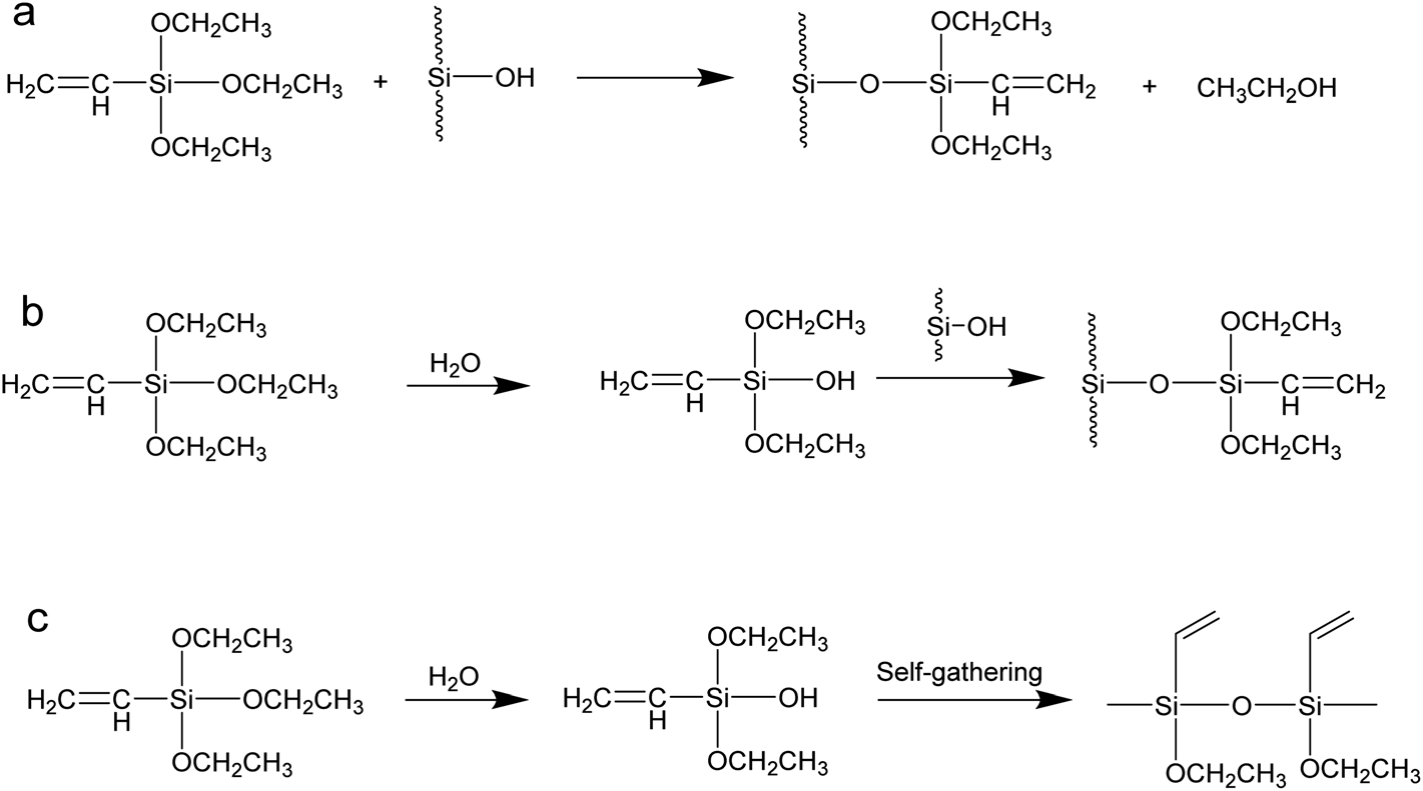
Reaction modes of coupling agent KH-151.

The effects of SG hydration degree on the grafting rate are shown in Fig. 16. Since silica gels with different hydration degrees have different reaction pathways during the grafting process, the hydration degree of SG has a great influence on the grafting rate. When the hydration degree of SG is 0, 3% and 5%, the grafting rate is relatively low, which is because when the hydration degree of silica gel is low, the grafting process is mainly carried out in a way that the alkoxy groups directly react with –OH on the surface of the silica gel, namely condensation of different functional groups, as shown in Fig. 17a.^44,45^ Compared with the condensation of same functional group, the condensation reaction of different functional groups has a higher energy barrier, higher reaction conditions and lower product yield.^46^ When the hydration degree of SG is high, the grafting reaction mainly proceeds in the manner of hydrolysis and condensation of same functional group, as shown in Fig. 17b. Since the alkoxy hydrolysis process and the –OH dehydration and condensation require lower reaction conditions, the reaction is more likely to occur, so the grafting rate is higher. When the hydration degree exceeds 10%, the water content in the reaction system is too large, the coupling agent hydrolysis degree is significantly increased, and self-polymerization occurs in the manner of Fig. 17c to form macromolecules. However, it is difficult for macromolecules to enter the pores and react with –OH on the surface of the silica gel, so the grafting rate is reduced. According to the experimental results, when the SG hydration degree reaches about 10%, the grafting rate reaches optimal.

### Orthogonal test

The results of orthogonal test are shown in Table 3. Using extreme difference analysis to process the orthogonal results, the primary and secondary relationship of the three influencing factors was *A* > *C* > *B* (hydration degree > coupling agent dosage > reaction temperature), and the optimal factor combination was *A*_2_*B*_3_*C*_3_, of which hydration degree was 10%, coupling agent KH-151 dosage was 12 mL and reaction temperature was 80 °C. Under the above optimal preparation process, the test was repeated three times to obtain an average grafting rate of 91.03%.

**Table 3:** Analysis of orthogonal test results

Number	Factors			Grafting rate (%)
	(*A*) Hydration degree	(*B*) Temperature	(*C*) KH-151 dosage	
1	1	1	1	82.27
2	2	2	2	86.53
3	3	3	3	86.29
4	1	2	3	85.31
5	2	3	1	88.96
6	3	1	2	83.57
7	1	3	2	86.34
8	2	1	3	89.62
9	3	2	1	83.15
K_1_	253.92	255.46	254.38	
K_2_	265.11	254.99	256.44	
K_3_	253.01	261.59	261.22	
K̄_1_	84.64	85.15	84.79	
K̄_2_	88.37	84.99	85.48	
K̄_3_	84.34	87.19	87.07	
R	4.03	2.2	2.28	

In order to verify the analysis results of the range method, we use SPSS software to perform orthogonal test variance analysis. As shown in Table 4, it can be seen from the results of the analysis of variance that the hydration degree has the most significant impact on the grafting rate, while the effects of temperature and coupling agent concentration are relatively small. According to the comparison of *F* values, the primary and secondary relationship of the three single factors is *A* > *C* > *B* (hydration degree > coupling agent dosage > reaction temperature), which is consistent with the results of the range analysis method.

**Table 4:** Intersubjective effect test, dependent variable: grafting rate^a^

Source	III sum of squares	Degree of freedom	Mean square	*F*	Significance
Revised model	47.521^a^	6	7.920	5.627	0.159
Intercept	66 227.307	1	66 227.307	47 051.663	0.000
A	30.273	2	15.136	10.754	0.085
B	9.040	2	4.104	2.916	0.237
C	8.209	2	4.520	3.211	0.255
Error	2.815	2	1.408		
Total	66 277.643	9			
Revised total	50.336	8			

a *R* ^2^ = 0.944.

## Conclusions

This paper studies the graft modification mechanism of coupling agent vinyl triethoxysilane (KH-151) to macroporous silica gel. From the results of NMR and XPS, it can be seen that the coupling agent molecules have two connection types on the surface of silica gel, and the ratio of the two types is 43.51% and 56.49% respectively. The influence of the hydration degree of silica gel, the coupling agent dosage and the reaction temperature on the grafting rate was explored, and the optimal reaction conditions for the modification of macroporous silica gel were determined by coupling agent through orthogonal experiments. Under optimal reaction conditions, the average grafting rate of coupling agent vinyl triethoxysilane (KH-151) on macroporous silica gel is as high as 91.03%. This work has explored a new path for the in-depth, detailed and comprehensive research on the surface chemical modification of silica gel, and provided a theoretical basis for the chemical modification of the surface of silica gel based on coupling agents.

## Author contributions

Zheng Wang: investigation, methodology, verification, writing – original draft; Mei-chen Liu: resources; Zhi-yuan Chang: conceptualization, writing–review & editing, supervision; Hui-bo Li: conceptualization, resources, project administration, funding acquisition.

## Conflicts of interest

The authors declare that no competing interests.

## Supplementary Material
